# Mössbauer and Structure-Magnetic Properties Analysis of A*_y_*B_1−*y*_C_*x*_Fe_2−*x*_O_4_ (C=Ho,Gd,Al) Ferrite Nanoparticles Optimized by Doping

**DOI:** 10.3390/molecules28104226

**Published:** 2023-05-22

**Authors:** Qing Lin, Fang Yang, Qian Zhang, Kaimin Su, Huiren Xu, Yun He, Jinpei Lin

**Affiliations:** 1 College of Biomedical Information and Engineering, Hainan Medical University, Haikou 571199, China; 2 College of Physics and Technology, Guangxi Normal University, Guilin 541004, China

**Keywords:** cobalt ferrite, substituted, doping, structure, magnetic, Mössbauer

## Abstract

A*_y_*B_1−*y*_C_*x*_Fe_2−*x*_O_4_ (C=Ho,Gd,Al) ferrite powders have been synthesized by the sol-gel combustion route. The X-ray diffraction of the CoHo_*x*_Fe_2−*x*_O_4_ (*x =* 0*~*0.08) results indicated the compositions of single-phase cubic ferrites. The saturation magnetisation of CoHo_*x*_Fe_2−__*x*_O_4_ decreased by the Ho^3+^ ions, and the coercivity increased initially and then decreased with the increase of the calcination temperature. The Mössbauer spectra indicated that CoHo_*x*_Fe_2−__*x*_O_4_ displays a ferrimagnetic behaviour with two normal split Zeeman sextets. The magnetic hyperfine field tends to decrease by Ho^3+^ substitution owing to the decrease of the A–B super-exchange by the paramagnetic rare earth Ho^3+^ ions. The value of the quadrupole shift was very small in the CoHo_*x*_Fe_2−*x*_O_4_ specimens, indicating that the symmetry of the electric field around the nucleus is good in the cobalt ferrites. The absorption area of the Mössbauer spectra changed with increasing Ho^3+^ substitution, indicating that the substitution influences the fraction of iron ions at tetrahedral A and octahedral B sites. The X-ray diffraction of Mg_0.5_Zn_0.5_C_*x*_Fe_2−*x*_O_4_(C=Gd,Al) results confirmed the compositions of single-phase cubic ferrites. The variation of the average crystalline size and lattice constant are related to the doping of gadolinium ions and aluminum ions. With increasing gadolinium ions and aluminum ions, the coercivity increased and the saturation magnetization underwent a significant change. The saturation magnetization of AlMg_0.5_Zn_0.5_FeO_4_ ferrite reached a minimum value (*M_S_* = 1.94 mu/g). The sample exhibited ferrimagnetic and paramagnetic character with the replacement with Gd^3+^ ions, that sample exhibited paramagnetic character with the replacement with Al^3+^ ions, and the isomer shift values indicated that iron is in the form of Fe^3+^ ions.

## 1. Introduction

Ferrite is important in various magnetic applications because it has the properties of magnetic materials and insulators [1,2,3,4]. Among the ferrite spinel structures, cobalt ferrites (CoFe_2_O_4_) are the important magnetic and magnetostriction materials [5,6,7,8]. As a famous hard magnetic material, cobalt ferrite has been widely used for magnetoelastic sensor applications [9,10,11,12]. The distribution of cations between tetrahedron (A) and octahedron (B) ferrite and the magnetic field interaction will affect the structure and electrical and magnetic properties [13,14,15,16]. Rare earth ions considerably affect the magneto-crystal anisotropy of ferrite by substituting Fe^3+^ ions in ferrites with the rare earth ions of 4*f* elements, which exhibit a strong spin-orbital (3*d*–4*f*) coupling [17,18,19]. Particularly, small amounts of rare earth holmium (Ho) elements affect the magnetic properties of cobalt ferrites [20,21,22]. Lohar et al. [8] studied the structural and magnetic properties of CoFe_2_O_4_ nanoparticles doped with rare earth Ho^3+^ ions, where the saturation magnetisation of CoFe_2_O_4_ nanoparticles increased with Ho^3+^ substitution, owing to the Ho^3+^ ions having a larger magnetic moment of 10.6 μ_B_. However, in other studies [9,10], the decrease in saturation magnetisation with Ho^3+^ substitution is attributed to Ho being paramagnetic at room temperature, weakening exchange interactions. Panneer Muthuselvam et al. [11] synthesised CoFe_1.95_Ho_0.05_O_4_ spinel ferrite annealed at different temperatures, which showed a single domain and multi-domain behaviour at a critical annealing temperature of 1050 °C. Rare earth ions will have a great influence on the magnetocrystalline anisotropy of ferrite [23,24,25,26]. Mg-Zn ferrite is important in soft magnetic materials and semiconducting magnetic materials [27,28,29]. Because Mg-Zn ferrite has low eddy current losses, high resistivity and high cost effective, it is widely used in computer memory, recording heads and loading coils [30]. Mg-Zn ferrite has a well-known cubic spinel structure, where most of the Magnesium (Mg^2+^) ions are distributed over the octahedral (B) sites and Zinc ions (Zn^2+^) tend to occupy the tetrahedral (A) sites [31]. The structure and magnetic properties of the sample are affected by the ion distribution [32]. Mukhtar et al. [6] synthesized Pr-substituted Mg-Zn ferrites by the sol-gel method, and studied the application of samples in magnetic cores and high frequency materials. Herein, we have synthesised A*_y_*B_1−*y*_C_*x*_Fe_2−*x*_O_4_ (C=Ho,Gd,Al) via the sol-gel-combustion method and investigated the variation of structural and magnetic properties for the ferrite sample with rare earth element substitution.

## 2. Results and Analysis

### 2.1. X-ray Diffraction Characterisation

**Figure 1:**
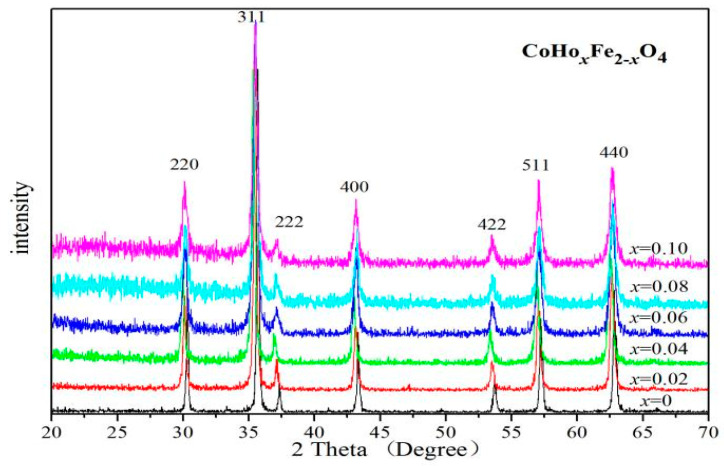
X-ray diffraction (XRD) patterns of CoHo_*x*_Fe_2−__*x*_O_4_ calcined at 800 °C.

Figure 1 shows the X-ray diffraction (XRD) patterns of ferrites CoHo_*x*_Fe_2−__*x*_O_4_ (*x* = 0–0.10) calcinated at 800 °C. The XRD patterns show that the CoHo_*x*_Fe_2−__*x*_O_4_ samples are single spinel-structure ferrites (JCPDS card no. 22-1086). No impurity peaks are observed in these XRD patterns. Owing to the ionic radius of Fe^3+^ ions (0.645 Å) being smaller than Ho^3+^ ions (0.901 Å) [9,11,12], when *x* ≤ 0.04, the lattice constants of CoHo_*x*_Fe_2−__*x*_O_4_ ferrite are larger than CoFe_2_O_4_ ferrite, as shown in Table 1. When *x* ≥ 0.06, the lattice parameter of CoHo_*x*_Fe_2−*x*_O_4_ ferrite is no longer increasing, possibly owing to the solubility limit of Ho^3+^ ions [9]. The average crystallite size of the CoHo_*x*_Fe_2−*x*_O_4_ samples estimated using the Debye–Scherrer formula [5,10,12] is between 21 and 55.6 nm. With Ho^3+^ doping, the decrease in the average crystallite size can be explained using the studies by others [14,15,16]. The RE^3+^ ions have an empty, half or filled 4*f* electron shell, such that rare earth Ho^3+^ ions substituted ferrites need more energy to grow grains and complete crystallisation.

The X-ray density of the sample was calculated by the following relationship [8,12,13]:

(1) $$\rho_{x}=\frac{8M}{Na^{3}}$$

where *a* is the lattice parameter, *N* is Avogadro’s constant, and *M* is the relative molecular mass.

Table 1 shows that the lattice parameter also tends to increase when the relative molecular mass increases with Ho^3+^ substitution. So, according to Formula (1), the X-ray density tends to increase with Ho^3+^ substitution because of the increase in the relative molecular mass.

**Figure 2:**
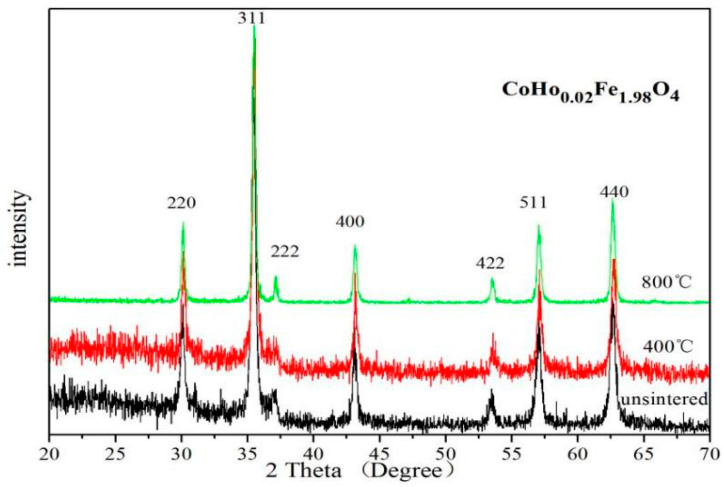
XRD patterns of CoHo_0.02_Fe_1.98_O_4_ un-sintered and sintered at 400 °C and 800 °C.

The XRD patterns of the un-sintered and sintered CoHo_0.02_Fe_2−__*x*_O_4_ samples are single spinel-structured ferrites, as shown in Figure 2. No impurity peaks were found in these XRD patterns. The breadth of XRD lines decreases with increasing heat treatment temperatures. As shown in Table 2, the X-ray density and the lattice parameter have no noticeable changes, and the average crystallite size of CoHo_0.02_Fe_1.98_O_4_ increases with increasing calcination temperature. Unlike in this study, the reported XRD diffraction peaks of CoFe_2_O_4_ calcined at a low temperature are not very sharp [4,14]. This indicates that the sample has good crystallinity without calcination.

**Figure 3:**
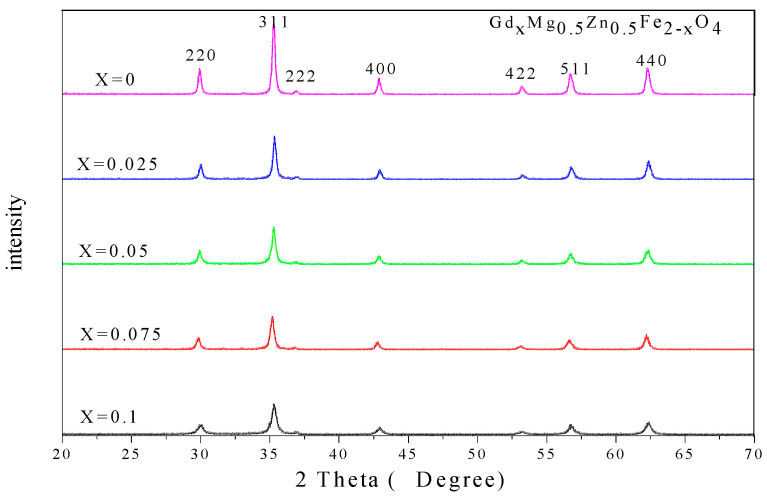
XRD patterns of Gd_*x*_Mg_0.5_Zn_0.5_Fe_2−*x*_O_4_ sintered at 800 °C.

**Figure 4:**
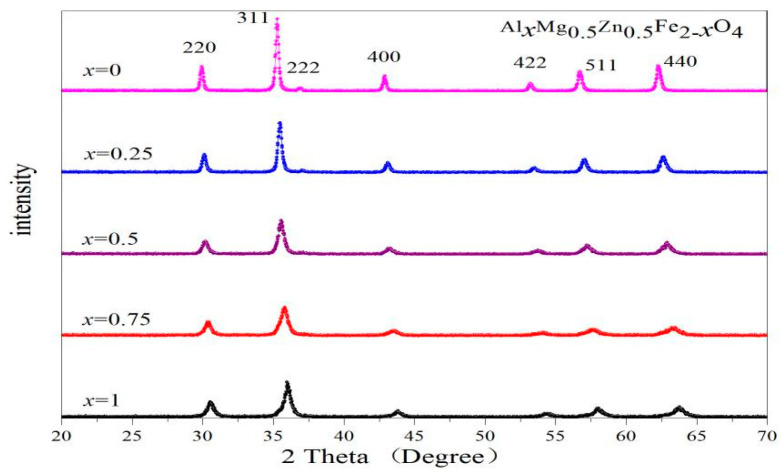
XRD patterns of Al_*x*_Mg_0.5_Zn_0.5_Fe_2−*x*_O_4_ sintered at 800 °C.

Figure 3 and Figure 4 show the X-ray diffraction (XRD) characterization of ferrites Gd_*x*_Mg_0.5_Zn_0.5_Fe_2−*x*_O_4_ and Al_*x*_Mg_0.5_Zn_0.5_Fe_2−*x*_O_4_ (*x* = 0~0.1). No impurity peaks were found in these XRD patterns. Table 3 indicates that the Gd_0.075_Mg_0.5_Zn_0.5_Fe_1.925_O_4_ ferrite has the maximum lattice constant value, due to the radius of Fe^3+^ ions being smaller than the radius of Gd^3+^ ions [30,33]. With the doping of rare earth Gd^3+^ ions, the lattice constant does not increase monotonously, due to the larger rare earth ionic radius leading to the lattice distortion of Mg-Zn ferrite [31,32]. Due to the radius of Al^3+^ ions being smaller than Fe^3+^ ions [34], the lattice constant of Al_*x*_Mg_0.5_Zn_0.5_Fe_2−*x*_O_4_ ferrite is smaller than Mg_0.5_Zn_0.5_Fe_2_O_4_ ferrite, and are shown in Table 4.

With the investigated samples Gd_*x*_Mg_0.5_Zn_0.5_Fe_2−*x*_O_4_ (x = 0~0.1), the average crystallite size estimated by the Debye-Scherrer formula is between 21.4 nm and 37.7 nm [29,30]. Additionally, the average crystallite size of Al_*x*_Mg_0.5_Zn_0.5_Fe_2−*x*_O_4_ (x = 0~0.1) is between 15.8 nm and 37.7 nm. The XRD density increases with Gd^3+^ substitution from Table 3. The doping of rare earth Gd ions will cause the relative molecular mass to increase, and the lattice constant of Mg-Zn ferrite has no significant change. Therefore, the increases in XRD density of Gd_*x*_Mg_0.5_Zn_0.5_Fe_2−*x*_O_4_ is attributed to the relative molecular mass increase. Moreover, the decreases in XRD density of Al_*x*_Mg_0.5_Zn_0.5_Fe_2−*x*_O_4_ are attributed to the fact that the relative molecular mass decreases.

**Figure 5:**
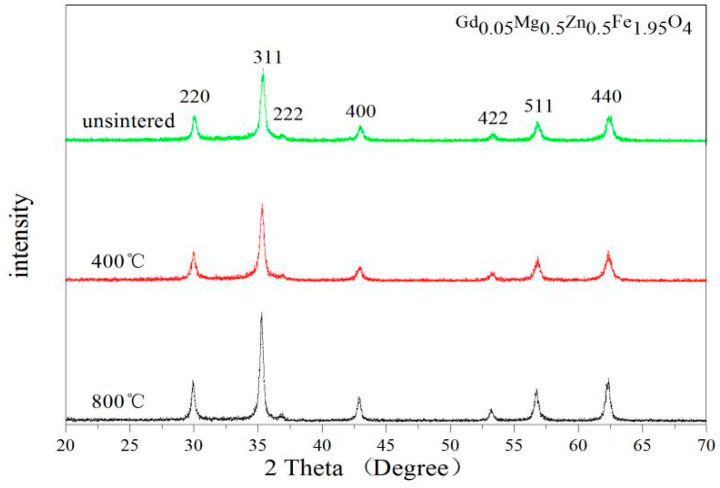
XRD patterns of Gd_0.05_Mg_0.5_Zn_0.5_Fe_1.95_O_4_ sintered at different temperatures.

**Figure 6:**
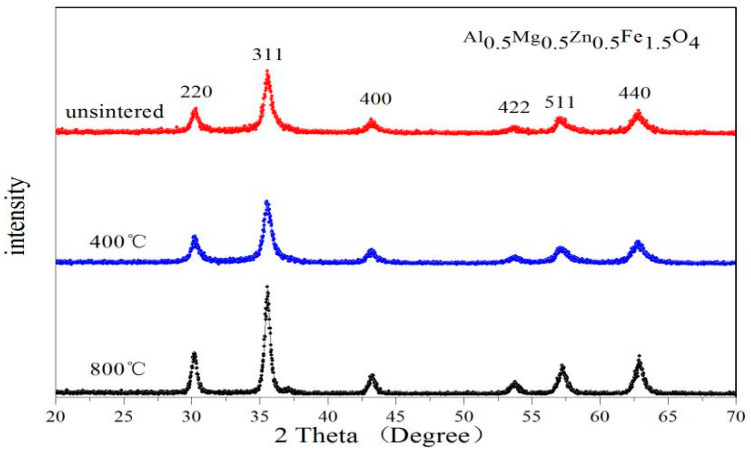
XRD patterns of Al_0.5_Mg_0.5_Zn_0.5_Fe_1.5_O_4_ sintered at different temperatures.

Figure 5 and Figure 6 are the XRD patterns of Gd_0.05_Mg_0.5_Zn_0.5_Fe_1.95_O_4_ and Al_0.5_Mg_0.5_Zn_0.5_Fe_1.5_O_4_ sintered at different temperatures. No impurity peaks were found in these XRD patterns. The breadth of XRD lines trends to decrease with increasing heat treatment temperatures. From Table 5 we know that the lattice constant and average crystallite size increases, and the XRD Density decreases with increasing the sintered temperature for the Gd_0.05_Mg_0.5_Zn_0.5_Fe_1.95_O_4_. For the Al_0.5_Mg_0.5_Zn_0.5_Fe_1.5_O_4,_ the lattice parameter decreases and the X-ray density increases, and average crystallite size is trend to increase with increasing the sintering temperature from Table 6. In other literature studies [35], the diffraction peaks of XRD is not very sharpness for CoGd_0.1_Fe_1.9_O_4_ calcined at low temperature, the diffraction peaks of XRD is very sharpness for Gd_0.05_Mg_0.5_Zn_0.5_Fe_1.95_O_4_ and Al_0.5_Mg_0.5_Zn_0.5_Fe_1.5_O_4_ without burning. This indicates that the sample has good crystallinity without sintering.

### 2.2. Scanning Electron Microscopy (SEM)

**Figure 7:**
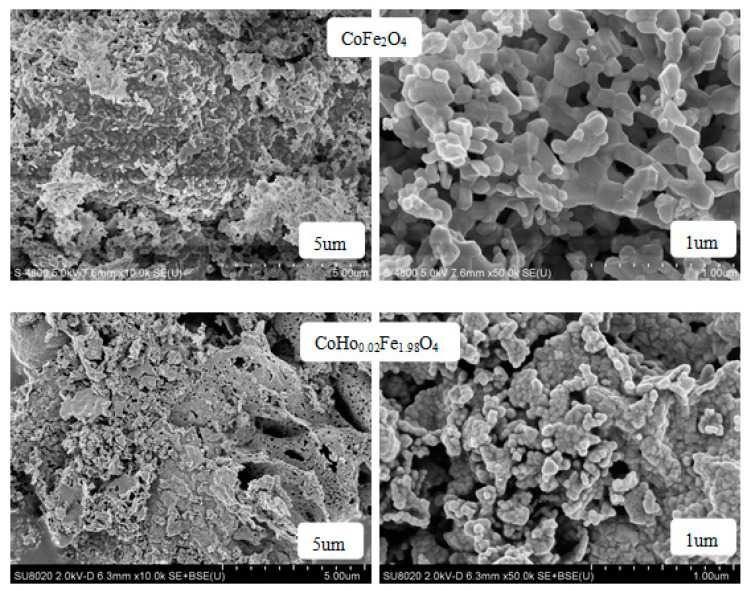
SEM images of CoFe_2_O_4_ and CoHo_0.02_Fe_1.98_O_4_ sintered at 800 °C.

Figure 7 shows the SEM images of CoHo_*x*_Fe_2−__*x*_O_4_ (*x* = 0, 0.02) annealed at 800 °C. As shown in Figure 3, the sample is well-crystallised, and the grain size is almost uniform. With Ho^3+^ substitution, some cobalt ferrite particles are agglomerated, possibly owing to the magnetic interactions between CoHo_*x*_Fe_2−__*x*_O_4_ particles [15]. 

**Figure 8:**
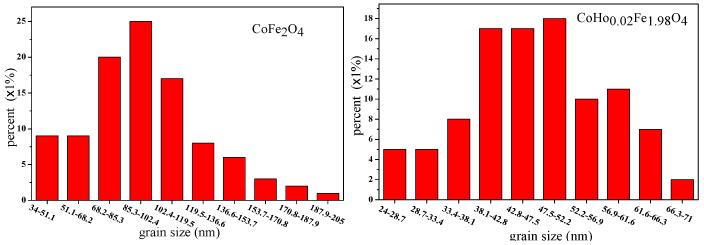
Grain size distribution of CoFe_2_O_4_ and CoHo_0.02_Fe_1.98_O_4_ calcined at 800 °C.

Figure 8 is the grain size distribution of CoFe_2_O_4_ and CoHo_0.02_Fe_1.98_O_4_. Using a statistical method, the calculated average grain size of CoFe_2_O_4_ and CoHo_0.02_Fe_1.98_O_4_ are ~96.26 and ~47.15 nm, respectively. The grain size of ferrite samples is less than 100 nm, indicating that they are nanoparticles. It is slightly larger than the average crystallite size determined using an X-ray, suggesting that each ferrite particle comprises several crystallites [16,17].

**Figure 9:**
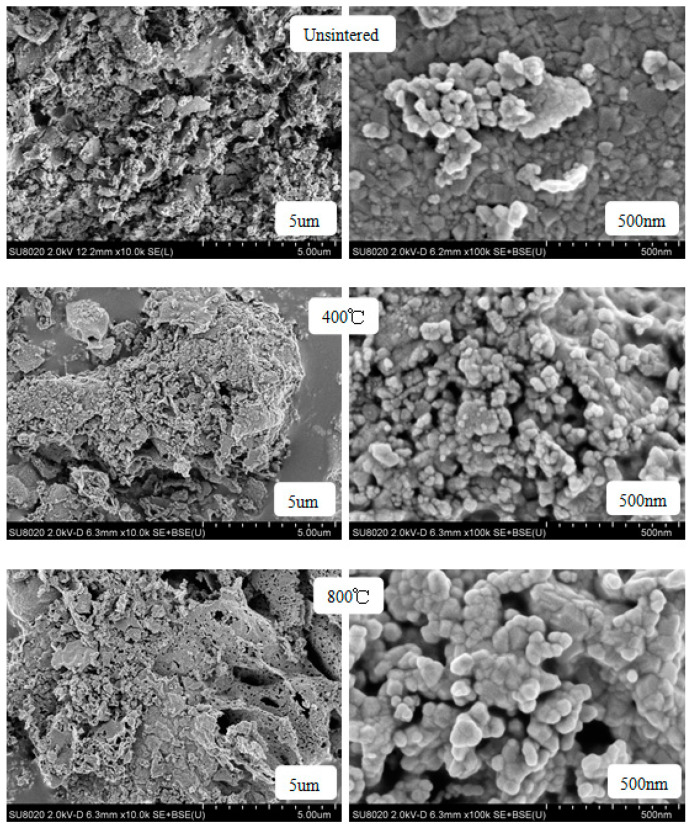
SEM images of CoHo_0.02_Fe_1.98_O_4_ un-sintered and sintered at 400 °C and 800 °C.

The SEM images of CoHo_0.02_Fe_1.98_O_4_ un-sintered and sintered at 400 °C and 800 °C are shown in Figure 9. As the heat treatment temperature increased, the crystallinity of the sample was improved, and the grain size increased. But the ferrite powders have a good crystallinity without heat treatment, consistent with the XRD analysis. Some cobalt ferrite particles are agglomerated with the increasing heat treatment temperature, owing to the magnetic interactions between CoHo_0.02_Fe_1.98_O_4_ particles [15], but the degree of agglomeration is small.

**Figure 10:**
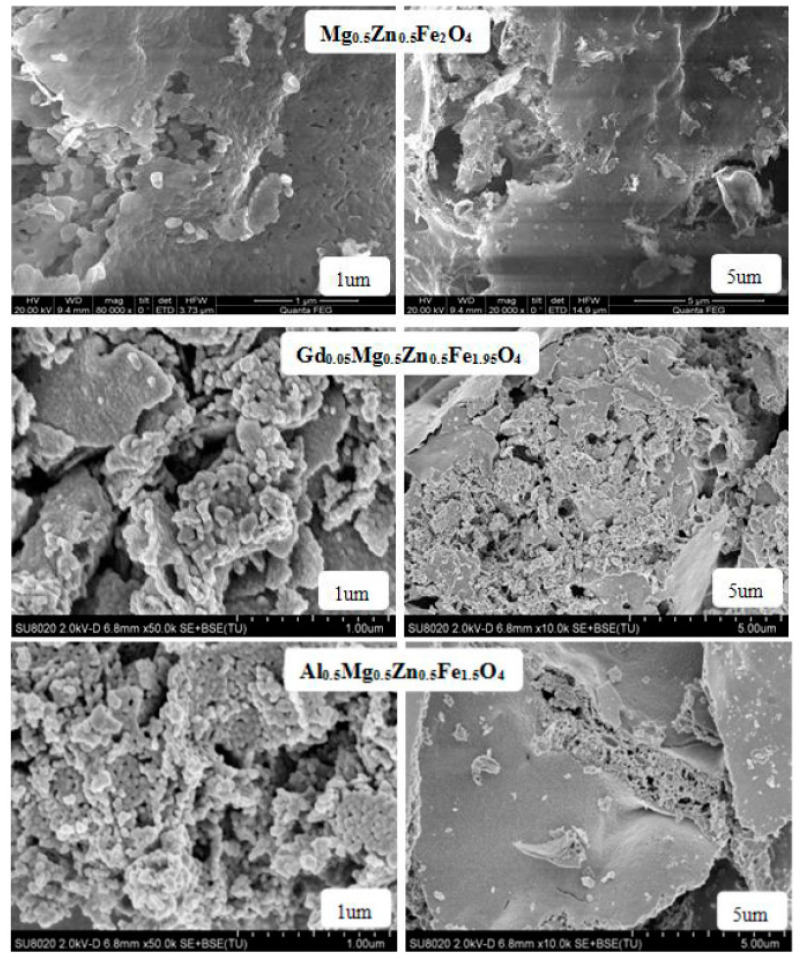
SEM micrographs of Mg_0.5_Zn_0.5_Fe_2_O_4_, Gd_0.05_Mg_0.5_Zn_0.5_Fe_1.95_O_4_ and Al_0.5_Mg_0.5_Zn_0.5_Fe_1.5_O_4_ sintered at 800 °C.

**Figure 11:**
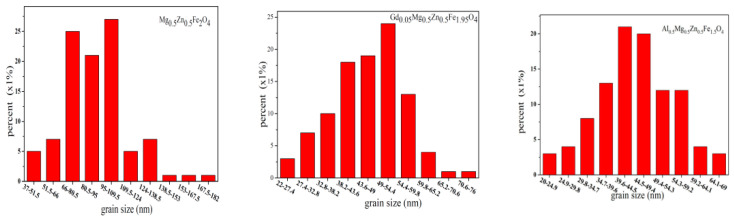
Histogram of grain size distribution of Mg_0.5_Zn_0.5_Fe_2_O_4_, Gd_0.05_Mg_0.5_Zn_0.5_Fe_1.95_O_4_ and Al_0.5_Mg_0.5_Zn_0.5_Fe_1.5_O_4_ sintered at 800 °C.

Figure 10 are the SEM images of Gd_0.05_Mg_0.5_Zn_0.5_Fe_1.95_O_4_ and Al_0.5_Mg_0.5_Zn_0.5_Fe_1.5_O_4_ sintered 800 °C. The sample is well crystallized, and the grain size is almost uniform. With the Gd^3+^ ions and Al^3+^ ions substituting, some particles of cobalt ferrite are agglomerated [21]. Figure 11 are the grain size histogram of Mg_0.5_Zn_0.5_Fe_2_O_4_, Gd_0.05_Mg_0.5_Zn_0.5_Fe_1.95_O_4_ and Al_0.5_Mg_0.5_Zn_0.5_Fe_1.5_O_4_. The average grain size is approximately 90.74nm, 46.11 nm and 44.84nm, respectively, and by a statistical method, the aluminum ion doping can make the grain size of the sample smaller. The grain size of ferrite samples is less than 100 nanometers, indicating that they are nanoparticles. However, they are slightly bigger than average crystallite size for an X-ray. Therefore, each particle is made up of several crystallites [17].

### 2.3. Magnetic Analysis

**Figure 12:**
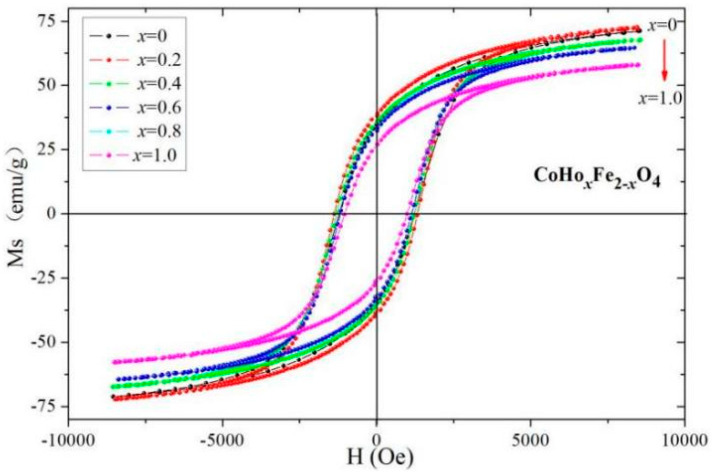
Hysteresis loops of CoHo_*x*_Fe_2−__*x*_O_4_ calcined at 800 °C.

Figure 12 shows the RT (at 295 K) magnetic hysteresis curve of CoHo_*x*_Fe_2−__*x*_O_4_ measured at 295 K. The magnetisation of CoHo_*x*_Fe_2−__*x*_O_4_ (*x* = 0–0.10) nearly reaches saturation at 10,000 Oe. The saturation magnetisation (*M_S_*) decreases with Ho^3+^ substitution, as shown in Table 7, which could be calculated according to the following relation [8,18]:

(2) $$M_{S}=\frac{5585\times n_{B}}{M}$$

where *n_B_* is the Bohr magneton and *M* is the molecular mass. The relative molecular mass of CoHo_*x*_Fe_2−__*x*_O_4_ increases as Ho^3+^ is replaced. The Ho^3+^, Co^2+^ and Fe^3+^ ions are 10.6, 3 and 5 μ_B_ [8], respectively. Ho exhibits para-magnetism at 295 K [9,10]. Ho^3+^ ions preferred to occupy B sites owing to the large Ho^3+^ ionic radius. Thus, the magnetic moment of the octahedral B site will decrease as the iron is replaced by holmium; this combined with the Néel theory, the total magnetic moment (*n_B_*) decreases. Owing to the increased *M* and the decreased *n_B_* with the substitution of Ho^3+^ ions, *M_S_* decreases according to the Formula (2). These results are consistent with other literature reports [9,20].

However, Table 7 shows that *M_S_* increases with Ho^3+^ concentration for *x* ≤ 0.02 because of a small amount of paramagnetic Ho^3+^ ions occupying A sites. The coercivity (*H*_C_) variation of cobalt ferrite with the substitution of Ho^3+^ ions can be explained as follows. The rare earth Ho^3+^ ions have substantial magneto-crystalline anisotropy [8,19,20]. *H*_C_ does not increase monotonously with the doping of rare earth Ho^3+^ ions because *H*_C_ is also related to the factors such as crystallinity and crystallite size [21].

**Figure 13:**
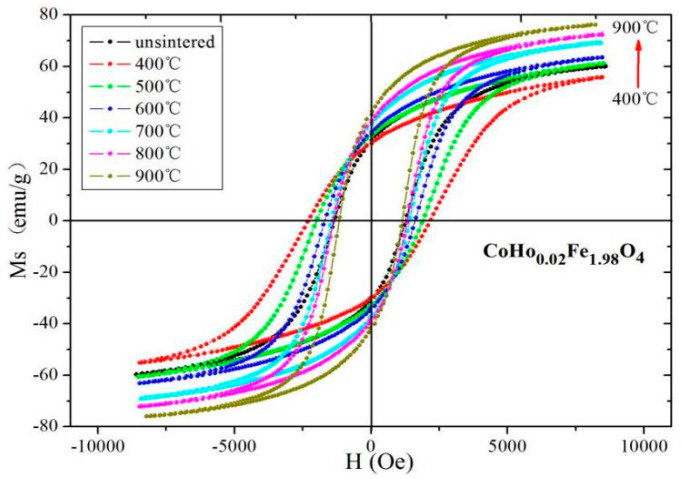
Hysteresis loops of CoHo_0.02_Fe_1.98_O_4_ at different sintering temperatures.

**Figure 14:**
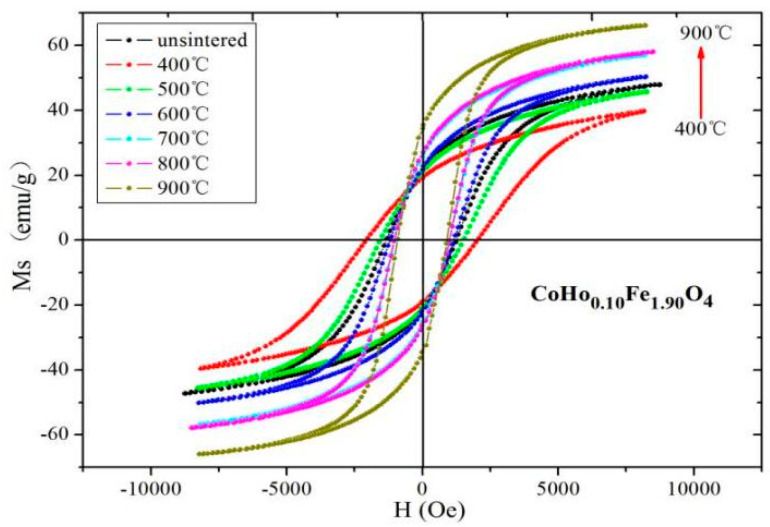
Hysteresis loops of CoHo_0.10_Fe_1.90_O_4_ at different sintering temperatures.

Magnetic hysteresis loops of CoHo_0.02_Fe_1.98_O_4_ and CoHo_0.10_Fe_1.90_O_4_ at different sintering temperatures are shown in Figure 13 and Figure 14, respectively. After annealing at 900 °C, CoHo_0.02_Fe_1.98_O_4_ and CoHo_0.10_Fe_1.90_O_4_ have the maximum *M_S_* value because particle size increases with the sintering temperature [22]. 

The *M_S_* of CoHo_0.02_Fe_1.98_O_4_ and CoHo_0.10_Fe_1.90_O_4_ decrease after annealing at 400 °C because the un-sintered sample may have a good crystallinity, as determined by XRD and SEM. Table 8 and Table 9 show that *M_S_* increases with Ho^3+^ concentration for *x* = 0.02 and *x* = 0.10. The *M_S_* of CoHo_0.02_Fe_1.98_O_4_ is larger than that of CoHo_0.10_Fe_1.90_O_4_, owing to the small amount of paramagnetic Ho^3+^ occupying A sites and the decreasing of the magnetic moment at the tetrahedral A site as the iron is replaced by holmium. According to Néel theory, the paramagnetic Ho^3+^ occupying A sites will increase *M_S_*. The *H*_C_ of CoHo_0.02_Fe_1.98_O_4_ and CoHo_0.10_Fe_1.90_O_4_ initially increases, decreasing as the sintering temperature increases. *H*_C_ is related to grain size (*D*), and their relationship is *H*_C_ = *g* − *h*/*D*^2^ (single domain region) and *H*_C_ = *a* + *b*/*D* (multi-domain region). According to the above-mentioned SEM analysis, the particle size increases with increasing calcination temperature, so the *H*_C_ increases initially and then decreases as the annealing temperature increases.

**Figure 15:**
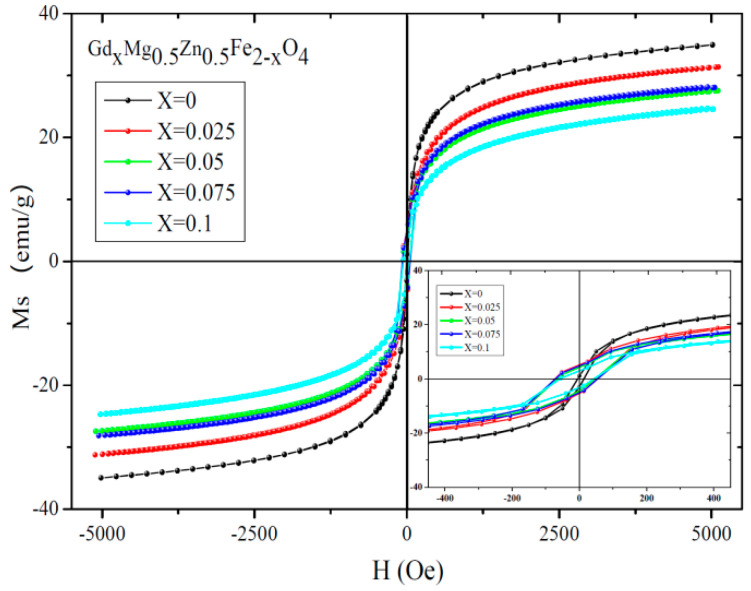
Hysteresis loops of Gd_*x*_Mg_0.5_Zn_0.5_Fe_2−*x*_O_4_ sintered at 800 °C.

**Figure 16:**
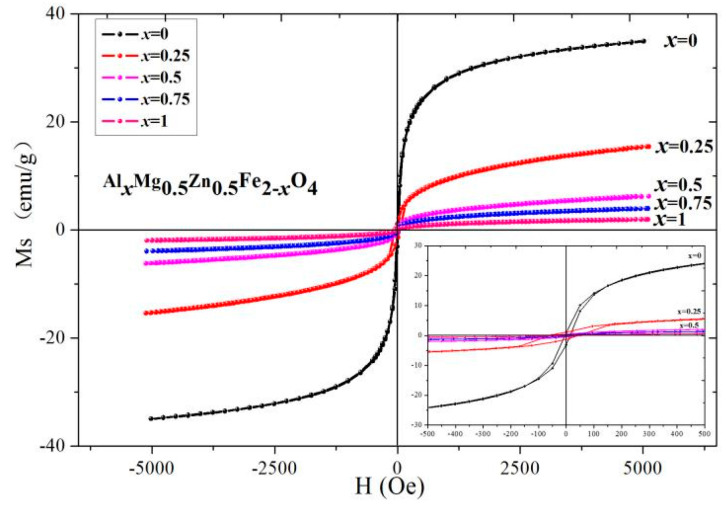
Hysteresis loops of Al_*x*_Mg_0.5_Zn_0.5_Fe_2−*x*_O_4_ sintered at 800 °C.

Figure 15 and Figure 16 show the magnetic hysteresis curve of Gd_*x*_Mg_0.5_Zn_0.5_Fe_2−*x*_O_4_ measured at 295 K. The magnetization nearly reaches saturation at 5000 Oe. The saturation magnetization decreases with the substitution of Gd^3+^ ions and Al^3+^ ions from Table 10 and Table 11. As Gd^3+^ replaces, the relative molecular mass of Gd_*x*_Mg_0.5_Zn_0.5_Fe_2−*x*_O_4_ increases. The magnetic moments of rare earth ions generally come from localized 4f electrons in solids, and the magnetic ordering temperature is low, and the Gd rare-earth element has the Curie temperature of 293.2 K, close to RT(295 K) [36,37]. The magnetic dipole orientation is disordered at 295 K, and the rare-earth ion doping has no contribution to magnetization. Therefore, the Gd^3+^ ions are considered as non-magnetic ions at 295 K [38]. Moreover, the magnetic moment of Fe^3+^ ions is 5 *μ_B_* [21].

Due to the relative molecular mass increase and the magnetic moment *n_B_* decrease with the doping of Gd^3+^ ions, the saturation magnetization decreases. Therefore, the magnetic moment of the octahedral B site will decrease as the iron is replaced by gadolinium and according to Neel theory the total magnetic moment *n_B_* will decrease. For the Al_*x*_Mg_0.5_Zn_0.5_Fe_2−*x*_O_4_, as the Al^3+^ are replaced the relative molecular mass and the total magnetic moment *n_B_* decreases, and the influence of the magnetic moment is greater than the relative molecular mass, so the saturation magnetization decreases. The coercivity of Gd_*x*_Mg_0.5_Zn_0.5_Fe_2−*x*_O_4_ is larger than that of the Mg_0.5_Zn_0.5_Fe_2_O_4_ from Table 10. The variation of coercivity with Gd^3+^ ions substituted MgZn ferrite can be explained as follows. Rare earth ions (Gd^3+^) have stronger magnetocrystalline anisotropy [33,39].

**Figure 17:**
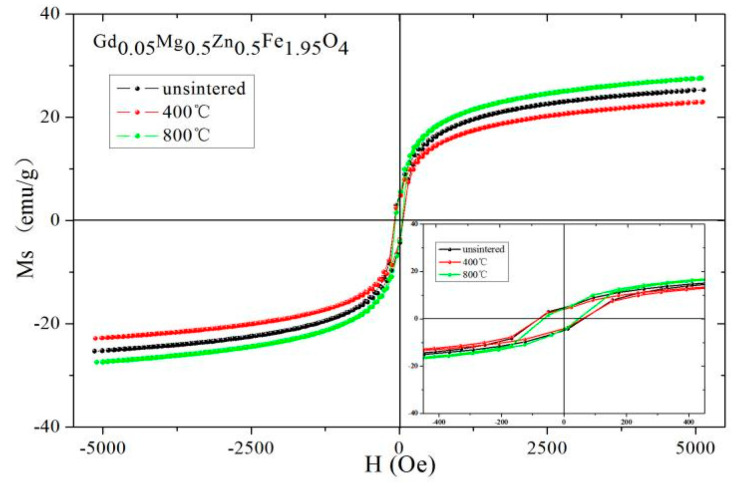
Hysteresis loops of Gd_0.05_Mg_0.5_Zn_0.5_Fe_1.95_O_4_ sintered at different temperatures.

Magnetic hysteresis loops of Gd_0.05_Mg_0.5_Zn_0.5_Fe_1.95_O_4_ at different sintering temperatures are shown in Figure 17. Table 12 indicates that Gd_0.05_Mg_0.5_Zn_0.5_Fe_1.95_O_4_ after annealing at 800 °C has the maximum value for saturation magnetization, because particle size increases with an increase in sintering temperature [29]. The coercivity of Gd_0.05_Mg_0.5_Zn_0.5_Fe_1.95_O_4_ decreases as sintering temperature increases. The coercivity (*H*_C_) is related to grain size (*D*), and their relationship is *H*_C_ = *g* − *h*/*D*^2^ (single domain region) and *H*_C_ = *a* + *b*/*D* (multidomain region) [30]. The coercivity increases with grain diameter increases in the single domain region, and the coercivity decreases as the grain size increases in the multidomain region. The grain size of Gd_0.05_Mg_0.5_Zn_0.5_Fe_1.95_O_4_ sintered at different temperatures is the multidomain region, so the coercivity decreases as annealing temperature increases.

### 2.4. Mössbauer Spectroscopy

**Figure 18:**
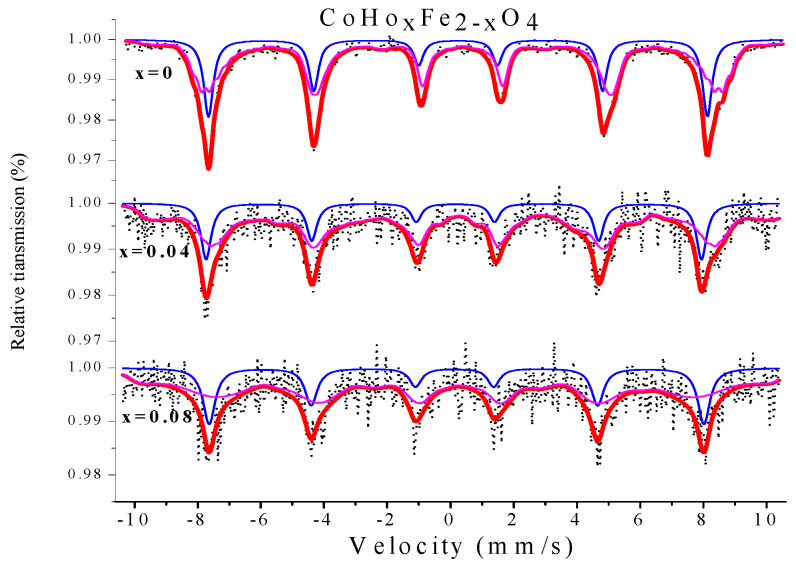
Mössbauer spectra of CoHo_*x*_Fe_2−__*x*_O_4_ calcined at 800 °C.

Figure 18 shows the Mössbauer spectra of CoHo_*x*_Fe_2−__*x*_O_4_ (*x* = 0, 0.04 and 0.08) polycrystalline ferrite powders measured at room temperature. All Mössbauer spectra were fitted using the Mösswinn 3.0 programme, and the spectra consist of two split Zeeman sextets, which indicates that the samples are ferrimagnetic. The six-line magnetic pattern with a larger isomer shift (I.S.) corresponds to the iron ion at position B, while the other six-line peak corresponds to the iron ion at position A [23,24]. As shown in Table 13, the changes in I.S. values are relatively small with the effects of Ho^3+^ substitution, and Ho^3+^ doping has no apparent impact on the *s*-electron charge distribution of Fe nuclei [24]. According to other reports, the I.S. values of Fe^3+^ ions are 0.1–0.5 mm/s [25,26]. From Table 13, the I.S. values indicate that the iron in the samples of this study is in the form Fe^3+^. Table 13 shows that the magnetic hyperfine field (H) tends to decrease by Ho^3+^ substitution, owing to the decrease of the A–B super-exchange by the paramagnetic rare-earth Ho^3+^ ions [27,28]. The value of the quadrupole shift is very small in the specimens of CoHo_*x*_Fe_2−__*x*_O_4_, indicating that the symmetry of the electric field around the nucleus is good in the cobalt ferrites. The absorption area of Mössbauer spectra for CoHo_*x*_Fe_2−__*x*_O_4_ has changed with increasing Ho substitution, which indicates that Ho^3+^ substitution influences the Fe^3+^ fraction at tetrahedral A and octahedral B sites.

**Figure 19:**
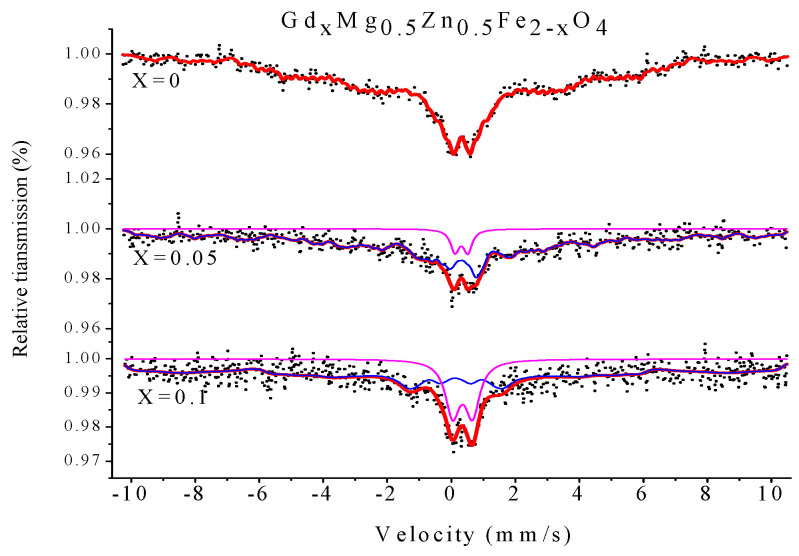
Mössbauer spectra of Gd_*x*_Mg_0.5_Zn_0.5_Fe_2−*x*_O_4_ sintered at 800 °C.

**Figure 20:**
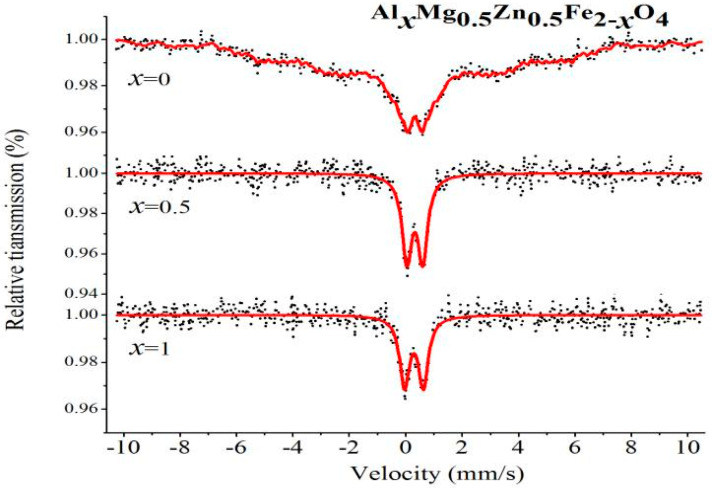
Mössbauer spectra of Al_*x*_Mg_0.5_Zn_0.5_Fe_2−*x*_O_4_ sintered at 800 °C.

Figure 19 and Figure 20 show the Mössbauer spectroscopy curve for Gd_*x*_Mg_0.5_Zn_0.5_Fe_2−*x*_O_4_ and Al_*x*_Mg_0.5_Zn_0.5_Fe_2−*x*_O_4_ polycrystalline ferrite powders measured at room temperature. For the Mg_0.5_Zn_0.5_Fe_2_O_4_, the spectra analyzed were a Zeeman-sextets-split, which shows features of relaxation effects. For the Gd_*x*_Mg_0.5_Zn_0.5_Fe_2−*x*_O_4_, when x = 0.05, 0.1, spectra of the samples fit a Zeeman-sextets-split and a central paramagnetic doublet [24,25,40]. According to other reports, the isomer shifts (I.S.) values of Fe^3+^ ions are 0.1–0.5 mm/s [27,36]. From Table 14 and Table 15, the isomer shifts values indicate that iron is in the form of Fe^3+^ ions. The value of the quadrupole shift is relatively small with effects of Gd^3+^ and Al^3+^ ions substitution, and Gd^3+^ doping has no obvious effect on the s-electron charge distribution of Fe nuclei [25,27]. The magnetic hyperfine field of the Zeeman-split sextet spectra decreases with rare earth Gd^3+^ ions and Al^3+^ ions substitution.

## 3. Experimental

A*_y_*B_1−*y*_C_*x*_Fe_2−*x*_O_4_ (C=Ho,Gd,Al) ferrite powders were synthesised via the sol-gel- combustion route. The synthetic raw materials of the sample are analytically pure nitrates (Co(NO_3_)_2_·6H_2_O, Fe(NO_3_)_3_·9H_2_O,Mg(NO_3_)_2_·6H_2_O, Zn(NO_3_)_2_·6H_2_O, Gd(N O_3_)_3_·9H_2_O, Al(NO_3_)_3_·9H_2_O and Ho(NO_3_)_3_·6H_2_O), ammonia (NH_3_·H_2_O) and citric acid (C_6_H_8_O_7_·H_2_O). Deionised water was added to form separate solutions of citric acid and metal nitrates. Then, citric acid and metal nitrates were mixed while adjusting the pH value at ~7 with ammonia. This solution mixture was kept on a thermostat water bath at 80 °C while electrically stirring the solution until it became a dried gel. The gel was further dried in the drying oven at 120 °C and ignited in the air with a small amount of alcohol as an oxidant. After spontaneous combustion, the material was annealed in the muffle furnace according to the specific temperature (400–900 °C). The crystalline structure was investigated by X-ray diffraction (D/max-2500V/PC, Rigaku, Tokyo, Japan). The micrographs were obtained by scanning electron microscopy (NoVa^TM^ Nano SEM 430, Diamond Bar, CA, USA). The Mössbauer spectrum was performed at room temperature, using a conventional Mössbauer spectrometer (Fast Com Tec PC-mossII, Oberhaching, Germany). Magnetization measurements were carried out with vibrating sample magnetometer (JDAW-2000D, Changchun INPRO Magneto-electric Technology Co., Ltd., Changchun, China) at room temperature.

## 4. Conclusions

A*_y_*B_1*−y*_C_*x*_Fe_2−*x*_O_4_ (C=Ho,Gd,Al) ferrite powders were synthesised via the sol-gel- combustion route. The XRD results show that CoHo_*x*_Fe_2−__*x*_O_4_ are single spinel-structured ferrites, and the CoHo_0.02_Fe_1.98_O_4_ samples calcined at different temperatures have good crystallinity. The SEM images further confirmed that the sample was well-crystallised, the grain was distributed homogeneously, and the sample comprised nanoparticles. The RT Mössbauer spectroscopy of CoHo_*x*_Fe_2−__*x*_O_4_ displayed ferromagnetic behaviour. Mössbauer spectra of CoHo_0.02_Fe_1.98_O_4_ showed that the calcination temperature affected the magnetic properties. The magnetisation results showed that rare-earth ion doping affects magnetic parameters, such as coercivity and saturation magnetisation, so the magnetic properties of samples can be regulated by rare-earth ion doping. Mg_0.5_Zn_0.5_C_*x*_Fe_2−*x*_O_4_(C=Gd,Al) ferrite sintered at different temperatures have good crystallinity. The rare-earth ion and aluminum ion doping, and sintering process had an effect on magnetic parameters such as coercivity and saturation magnetization. The Mössbauer spectra showed that the sample exhibited ferrimagnetic and paramagnetic character with the replaced Gd^3+^ ions; that the sample exhibited paramagnetic character with the replaced Al^3+^ ions; and that the isomer shift values indicated that iron is in the form of Fe^3+^ ions.

## Figures and Tables

**Table 1:** XRD date of CoHo_*x*_Fe_2−*x*_O_4_ calcined at 800 °C.

Content (*x*)	Lattice Parameter (Å)	Average Crystallite Size (Å)	Density (g/cm^3^)
0	8.35497	556	5.3468
0.02	8.38601	350	5.3342
0.04	8.40681	308	5.3434
0.06	8.38132	229	5.4415
0.08	8.37719	249	5.4989
0.10	8.38928	221	5.5243

**Table 2:** XRD date of CoHo_0.02_Fe_1.98_O_4_ un-sintered and sintered at 400 °C, 800 °C.

Temperature (°C)	Lattice Parameter (Å)	Average Crystallite Size (Å)	Density (g/cm^3^)
Un-sintered	8.38379	249	5.3384
400	8.39279	293	5.3212
800	8.38601	350	5.3342

**Table 3:** XRD date of Gd_*x*_Mg_0.5_Zn_0.5_Fe_2−*x*_O_4_ sintered at 800 °C.

Content (*x*)	Lattice Constant (Å)	Average Crystallite Size (Å)	Density (g/cm^3^)
0	8.43133	377	4.8879
0.025	8.42094	344	4.9624
0.05	8.43047	301	5.0018
0.075	8.44593	290	5.0303
0.1	8.42142	214	5.1307

**Table 4:** XRD date of Al_*x*_Mg_0.5_Zn_0.5_Fe_2−*x*_O_4_ sintered at 800 °C.

Content (*x*)	Lattice Parameter (Å)	Average Crystallite Size (Å)	Density (g/cm^3^)
0	8.43133	377	4.8879
0.25	8.39110	290	4.7963
0.5	8.36480	202	4.6779
0.75	8.31315	158	4.5988
1.0	8.25874	179	4.5201

**Table 5:** XRD date of Gd_0.05_Mg_0.5_Zn_0.5_Fe_1.95_O_4_ sintered at different temperatures.

Temperature (°C)	Lattice Constant (Å)	Average Crystallite Size (Å)	Density (g/cm^3^)
Un-sintered	8.41517	228	5.0292
400 °C	8.41829	233	5.0236
800 °C	8.43047	301	5.0018

**Table 6:** XRD date of Al_0.5_Mg_0.5_Zn_0.5_Fe_1.5_O_4_ sintered at different temperatures.

Temperature (°C)	Lattice Parameter (Å)	Average Crystallite Size (Å)	Density (g/cm^3^)
Un-sintered	8.38226	165	4.6487
400 °C	8.36985	137	4.6694
800 °C	8.36480	202	4.6779

**Table 7:** Magnetic data for CoHo_*x*_Fe_2−*x*_O_4_ calcined at 800 °C.

Content (*x*)	*M_S_* (emu/g)	*H*_C_ (Oe)	M_r_ (emu/g)
0	70.58	1005	34.71
0.02	72.54	1351	37.77
0.04	67.58	1266	35.06
0.06	64.64	1158	31.90
0.08	61.82	1094	30.24
0.10	57.96	1016	26.28

**Table 8:** Magnetic data of CoHo_0.02_Fe_1.98_O_4_ at different sintering temperatures.

Temperature (°C)	*M_S_* (emu/g)	*H*_C_ (Oe)	M_r_ (emu/g)
Un-sintered	60.01	1301	29.98
400	55.80	2234	29.62
500	61.10	1944	32.37
600	63.52	1655	33.56
700	69.21	1426	36.91
800	72.54	1351	37.77
900	76.16	1152	40.99

**Table 9:** Magnetic data of CoHo_0.10_Fe_1.90_O_4_ at different sintering temperatures.

Temperature (°C)	*M_S_* (emu/g)	*H*_C_ (Oe)	M_r_ (emu/g)
Un-sintered	47.89	1290	20.68
400	39.75	2052	18.83
500	45.72	1533	20.42
600	50.30	1174	21.50
700	57.05	1014	25.13
800	57.96	1016	26.28
900	66.12	914	33.8

**Table 10:** Magnetic data for Gd_*x*_Mg_0.5_Zn_0.5_Fe_2−*x*_O_4_ sintered at 800 °C.

Content (*x*)	*M_S_* (emu/g)	*H*_C_ (Oe)	M_r_ (emu/g)
0	34.98	13.64	1.10
0.025	31.38	62.52	4.67
0.05	27.54	55.79	3.79
0.075	28.17	62.99	4.36
0.1	24.67	50.52	2.61

**Table 11:** Magnetic data for Al_*x*_Mg_0.5_Zn_0.5_Fe_2−*x*_O_4_ calcined at 800 °C.

Content (*x*)	*M_S_* (emu/g)	*H*_C_ (Oe)	M_r_ (emu/g)
0	34.98	13.64	1.10
0.25	15.39	50.37	1.38
0.5	6.26	53.56	0.54
0.75	3.96	50.37	0.33
1	1.94	59.06	0.16

**Table 12:** Magnetic data for Gd_0.05_Mg_0.5_Zn_0.5_Fe_1.95_O_4_ sintered at different temperatures.

Temperature (°C)	*M_S_* (emu/g)	*H*_C_ (Oe)	M_r_ (emu/g)
Un-sintered	25.29	71.92	4.39
400 °C	22.93	69.58	3.73
800 °C	27.54	55.79	3.79

**Table 13:** Mössbauer parameters of CoHo_*x*_Fe_2−*x*_O_4_ calcined at 800 °C.

Content (*x*)	Component	I.S. (mm/s)	Q.S. (mm/s)	H (T)	Γ (mm/s)	A_0_ (%)
0	Sextet (A)	0.237	−0.004	48.946	0.360	32.4
	Sextet (B)	0.375	−0.024	45.695	0.322	67.6
0.04	Sextet (A)	0.126	−0.050	48.613	0.387	22.2
	Sextet (B)	0.316	0.151	44.156	0.429	77.8
0.08	Sextet (A)	0.157	0.061	48.546	0.463	24.5
	Sextet (B)	0.224	−0.137	43.641	0.740	75.5

**Table 14:** Mössbauer parameters of Gd_*x*_Mg_0.5_Zn_0.5_Fe_2−*x*_O_4_ sintered at 800 °C.

Content (*x*)	Component	I.S. (mm/s)	Q.S. (mm/s)	H (T)	Γ (mm/s)	A_0_ (mm/s)
0	Sextet (B)	0.318	0.005	24.234	0.268	100
0.05	Sextet (B)	0.302	−0.173	30.350	0.334	93.1
	Doublet	0.301	0.409	-	0.327	6.9
0.1	Sextet (B)	0.116	−0.029	38.679	0.528	79.4
	Doublet	0.348	0.627	-	0.537	20.6

**Table 15:** Mössbauer parameters of Al_*x*_Mg_0.5_Zn_0.5_Fe_2−*x*_O_4_ sintered at 800 °C.

Content (*x*)	Component	I.S. (mm/s)	Q.S. (mm/s)	H (T)	Γ (mm/s)	A_0_ (mm/s)
0	Sextet (B)	0.318	0.005	24.234	0.268	100
0.5	Double	0.322	0.568	-	0.417	100
1	Double	0.292	0.672	-	0.414	100

## Data Availability

Not applicable.
